# Effectiveness, Tolerability, and Drug Survival of Risankizumab in a Real-World Setting: A Three-Year Retrospective Multicenter Study—IL PSO (ITALIAN LANDSCAPE PSORIASIS)

**DOI:** 10.3390/jcm13020495

**Published:** 2024-01-16

**Authors:** Luigi Gargiulo, Luciano Ibba, Piergiorgio Malagoli, Fabrizio Amoruso, Giuseppe Argenziano, Anna Balato, Federico Bardazzi, Martina Burlando, Carlo Giovanni Carrera, Giovanni Damiani, Paolo Dapavo, Valentina Dini, Chiara Franchi, Francesca Maria Gaiani, Giampiero Girolomoni, Claudio Guarneri, Claudia Lasagni, Francesco Loconsole, Angelo Valerio Marzano, Martina Maurelli, Matteo Megna, Diego Orsini, Francesca Sampogna, Massimo Travaglini, Mario Valenti, Antonio Costanzo, Alessandra Narcisi

**Affiliations:** 1 Dermatology Unit, IRCCS Humanitas Research Hospital, Rozzano, 20089 Milan, Italy; luciano.ibba@humanitas.it (L.I.); mario.valenti@hunimed.eu (M.V.); antonio.costanzo@hunimed.eu (A.C.); alessandra.narcisi@humanitas.it (A.N.); 2 Department of Biomedical Sciences, Humanitas University, Pieve Emanuele, 20089 Milan, Italy; 3 Department of Dermatology, Dermatology Unit Azienda Ospedaliera San Donato Milanese, 20097 Milan, Italy; dermapier@gmail.com (P.M.); fm.gaiani@gmail.com (F.M.G.); 4 Dermatology Unit, Azienda Ospedaliera di Cosenza, 87100 Cosenza, Italy; amorusofabrizio.g@gmail.com; 5 Dermatology Unit, University of Campania L. Vanvitelli, 80138 Naples, Italy; g.argenziano@gmail.com (G.A.); anna.balato@unicampania.it (A.B.); 6 Dermatology Unit, IRCCS Azienda Ospedaliero-Universitaria di Bologna, Policlinico S. Orsola Malpighi, 40138 Bologna, Italy; federico.bardazzi@unibo.it; 7 Department of Dermatology, Dipartimento di Scienze della Salute (DISSAL), University of Genoa, IRCCS Ospedale Policlinico San Martino, 16100 Genoa, Italy; martinaburlando@hotmail.com; 8 Dermatology Unit, Fondazione IRCCS Ca’ Granda Ospedale Maggiore Policlinico, 20122 Milan, Italy; carlo.carrera@policlinico.mi.it (C.G.C.); angelo.marzano@unimi.it (A.V.M.); 9 Department of Biomedical, Surgical and Dental Sciences, University of Milan, 20133 Milan, Italy; dottdamiani@yahoo.com; 10 Clinical Dermatology, IRCCS Ospedale Galeazzi-Sant’Ambrogio, 20157 Milan, Italy; franchi_c@libero.it; 11 Department of Biomedical Science and Human Oncology, Second Dermatologic Clinic, University of Turin, 10124 Turin, Italy; paolo.dapavo@gmail.com; 12 Dermatology Unit, Department of Clinical and Experimental Medicine Ospedale Santa Chiara, 11 Via Roma 67, 56126 Pisa, Italy; valentinadini74@gmail.com; 13 Department of Medicine, Section of Dermatology and Venereology, University of Verona, Piazzale A. Stefani 1, 37126 Verona, Italy; giampiero.girolomoni@univr.it (G.G.); maurelli.martina@gmail.com (M.M.); 14 Department of Biomedical and Dental Sciences and Morphofunctional Imaging, Unit of Dermatology, University of Messina, AOU Policlinico G. Martino, Via Consolare Valeria 1, 98125 Messina, Italy; claudio.guarneri@unime.it; 15 Dermatological Clinic, Department of Specialized Medicine, University of Modena, Via del Pozzo 71, 41121 Modena, Italy; lasacla65@gmail.com; 16 Department of Dermatology, University of Bari, Piazza Umberto I, 1, 70121 Bari, Italy; franciscus59@gmail.com; 17 Department of Pathophysiology and Transplantation, Università degli Studi di Milano, 20122 Milan, Italy; 18 Section of Dermatology, Department of Clinical Medicine and Surgery, University of Naples Federico II, 80131 Naples, Italy; mat24@libero.it; 19 UOC Clinical Dermatology—Dermatological Institute S. Gallicano, IRCCS, 00167 Rome, Italy; diegorsini@gmail.com; 20 Clinical Epidemiology Unit, Istituto Dermopatico dell’Immacolata (IDI), IRCCS, 00167 Rome, Italy; fg.sampogna@gmail.com; 21 U.O.S.D. Dermatologica—Centro per la Cura Della Psoriasi, Ospedale Perrino, 72100 Brindisi, Italy; mtmtravaglini@gmail.com

**Keywords:** psoriasis, risankizumab, real-world evidence, biological therapy

## Abstract

Background: Risankizumab is a humanized monoclonal antibody that selectively inhibits interleukin-23. It has been approved for moderate-to-severe plaque psoriasis and has shown efficacy and safety in clinical trials and real-world experiences. This study aimed to evaluate the long-term effectiveness, safety, and drug survival of risankizumab in a real-life setting. Materials and Methods: We included patients treated with risankizumab from January 2019 to February 2023. A Psoriasis Area and Severity Index score (PASI) was collected at weeks 0, 16, 28, 52, 104, and 156, when available. The occurrence of any adverse events was recorded at each visit. Results: We enrolled 1047 patients. At week 52, a ≥90% improvement in PASI was observed in 81.44% of patients, with a continuous improvement throughout the study (88.99% and 99.07% at weeks 104 and 156, respectively). After three years of treatment, all patients involving the scalp, palms/soles, and genitalia and 95% of patients with nail psoriasis achieved a complete or almost complete skin clearance. No significant safety findings were observed, and 90.73% of the patients were still on treatment after 36 months. Conclusions: This study supports the long-term effectiveness and safety of risankizumab in a real-world setting, even in patients involving difficult-to-treat areas.

## 1. Introduction

Psoriasis is a chronic inflammatory immune-mediated disease that affects up to 4% of the general population [1]. Clinically, psoriasis vulgaris manifests itself with erythematous scaly plaques and patches that most commonly involve the knees, elbows, scalp, and lumbosacral area, but it can affect the whole body’s surface [1]. It can present as a mild disease requiring topical treatments or as a moderate-to-severe disease requiring systemic therapies [2]. In particular, first-line therapies for the treatment of moderate-to-severe psoriasis are represented by phototherapy or conventional immunosuppressive drugs (i.e., cyclosporine and methotrexate) [3]. Biological therapies are the treatment of choice when conventional treatments are contraindicated or show inadequate efficacy [4]. In particular, several biologics have been approved during the last five years, targeting tumor necrosis factor-alpha (TNF-α) or interleukin (IL)-23, IL-12/23, or IL-17, which have been established as the pivotal cytokines in the pathogenesis of psoriasis [5,6,7].

IL-23 inhibitors include guselkumab, risankizumab, and tildrakizumab, the former two also being approved for psoriatic arthritis (PsA) [8]. In particular, risankizumab is an IgG1-humanized monoclonal antibody that has been approved for the treatment of moderate-to-severe plaque psoriasis after being evaluated in several clinical trials (UltIMMa-1, UltIMMa-2, IMMerge, IMMhance, IMMvent) showing superior efficacy compared with placebos, ustekinumab, secukinumab, and adalimumab [9,10,11,12]. The effectiveness of risankizumab has been confirmed by several real-life experiences [13,14]. Recently, our group published data on a “super-responder” profile of patients treated with risankizumab, including those who were bio-naïve and with a short disease history (defined as a medical history of psoriasis shorter than 2 years at the moment of the treatment with risankizumab) [13]. However, data on the long-term effectiveness and tolerability of risankizumab are still limited, especially regarding different subpopulations, such as patients with cardiometabolic comorbidities (arterial hypertension, hypercholesterolemia, cardiovascular disease, obesity, and type 2 diabetes mellitus), PsA, and the involvement of difficult-to-treat areas (including palms and soles, scalp, genitalia, and nails).

We report the results of a multicenter retrospective real-life study aimed to assess the effectiveness and safety of risankizumab through 156 weeks of treatment in 1047 patients affected by moderate-to-severe plaque psoriasis.

## 2. Materials and Methods

### 2.1. Study Design and Population

We collected data from the electronic databases of 17 Italian Dermatology Units from January 2019 to February 2023. We enrolled 1047 patients who had undergone a minimum of one follow-up visit. All patients received risankizumab following the Italian Guidelines for the management of plaque psoriasis [15]. Risankizumab was administrated according to the summary of product characteristics in patients with inadequate response or contraindications to systemic treatments [16]. Institutional review board approval was exempted for this study as its procedure did not deviate from good routine clinical practice. All patients gave written informed consent for the retrospective retrieval of anonymized data. The study was conducted in accordance with the Helsinki Declaration of 1964 and its later amendments.

### 2.2. Data Collection and Outcomes

At each dermatological examination, the Psoriasis Area and Severity Index score (PASI) was recorded. Baseline characteristics were age, gender, body mass index (BMI), comorbidities, previous exposure to biological drugs, and the involvement of difficult-to-treat areas (scalp, palms/soles, genitalia, and nails). For patients who did not attend the scheduled dermatological visits and performed the injection of risankizumab at home or skipped the dose, data from the last available visit were used using the last-observation-carried-forward analysis (a missing follow-up visit value is replaced by that subject’s previously observed value). The effectiveness of risankizumab was evaluated in terms of improvements of 90% and 100% in PASI score compared with baseline (PASI 90 and PASI 100, respectively). Data from the dermatological visits at weeks 16, 28, 52, 104, and 156 were analyzed. Drug survival of risankizumab after 36 months of treatment was also examined.

The effectiveness of risankizumab in terms of PASI 90 and PASI 100 was evaluated according to different variables, including BMI, cardiometabolic comorbidities, concomitant PsA, and the presence of difficult-to-treat areas after one, two, and three years. Moreover, we conducted additional analyses on patients with the involvement of difficult-to-treat areas who had at least one year of follow-up. Nine hundred sixteen patients were included in this cohort. We assessed the percentage of patients who achieved a site-specific Physicians Global Assessment (PGA) of 0 or 1 (clear or almost clear) after one, two, and three years.

At each visit, the occurrence of any adverse events (AEs) was recorded, including serious AEs and AEs leading to risankizumab discontinuation.

### 2.3. Statistical Analysis

The chi-square and Exact Fisher’s tests were used to analyze categorical variables, while continuous data were compared using Student’s *t*-test and Mann–Whitney U tests. We used one-way ANOVA or the Kruskal–Wallis test if the distributions were not normal to test the differences between more than two groups. A logistic regression analysis was performed for all variables with a probability value (*p*-value) of less than 0.2 in the univariate analysis. Odds ratios (ORs) and 95% confidence intervals (CIs) were reported. All patients treated with risankizumab were included in the drug survival analysis using Kaplan–Meier curves. The event date was chosen as the date the patient discontinued the drug by any cause. Time data were censored for patients who were still on treatment when the study was conducted and for patients lost to follow-up. The differences in drug survival between subgroups were assessed with the log-rank test. We also conducted Cox regression analysis to determine predictive variables for drug survival. Moreover, we evaluated the mean time to drug discontinuation for the patients who experienced a loss of effectiveness during the study. A *p*-value < 0.05 was considered significant. All graphs were generated using Graph Pad—PRISM 9, while the analyses were conducted using Microsoft Excel 2021 and STATA/SE 17.0 software.

## 3. Results

### 3.1. Patients’ Characteristics

We included 1047 patients from 17 Italian referral hospitals. Seven hundred five patients were males (67.44%), and their mean age was 51.37 years with a standard deviation (SD) of 14.95. Five hundred thirty-five of them (51.10%) had at least one cardio-metabolic comorbidity (CMD), including arterial hypertension, obesity, type 2 diabetes mellitus, hypercholesterolemia, and cardiovascular disease, and 12.13% had concomitant PsA. Data regarding the BMI were available for 944 patients: mean BMI was 28.05 (SD 5.66), and 27.12% of patients were classified as obese, 36.65% as overweight, and 36.23% as normal weight. They had a mean disease history of 16.03 years (SD 12.25), and 42.60% were previously treated with at least one biological drug. One hundred eighty-three patients (17.48%) had previously received adalimumab, seventy-seven (7.35%) were treated with etanercept, twenty-two (2.1%) with infliximab, and one (0.1%) with certolizumab. A significant percentage of our patients were previously exposed to anti-IL-17 drugs, as 153 (14.61%) of them had received secukinumab, 98 (9.36%) ixekizumab, and 38 (3.63%) brodalumab. Finally, 126 patients (12.03%) had previously failed ustekinumab, 24 (2.29%) guselkumab, and 15 tildrakizumab (1.43%). Slightly more than half of our cohort (59.50%) reported the involvement of at least one difficult-to-treat area. Additional baseline characteristics of our patients are shown in Table 1.

### 3.2. Risankizumab Effectiveness

**Figure 1:**
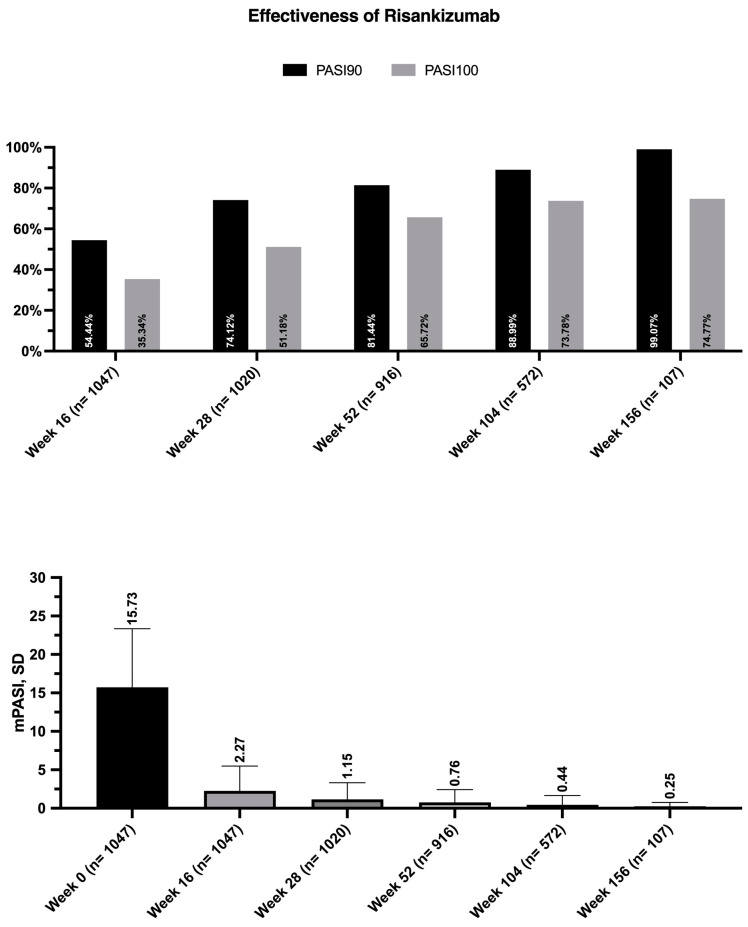
Effectiveness outcomes of risankizumab throughout the study period. PASI: Psoriasis Area and Severity Index; SD: standard deviation.

Throughout the study period, mean PASI decreased from 15.73 (SD 7.65) at baseline to 2.27 (3.22) at week 16, 1.15 (2.17) at week 28, and 0.76 (1.66) at week 52. Concerning the patients who completed two and three years of follow-up, their mean PASI scores were 0.44 (1.22) and 0.25 (0.51), respectively (Figure 1).

After 16 weeks, PASI 90 and PASI 100 were achieved by 54.44% and 35.34%, respectively. We noted sustained effectiveness over the course of the study, with 81.44% of patients achieving PASI 90 at week 52, 88.99% at week 104, and almost all patients (99.07%) after three years of treatment. Moreover, after one, two, and three years, 65.72%, 73.73%, and 74.77% of our patients achieved complete skin clearance, respectively (Figure 1).

### 3.3. Effectiveness of Risankizumab in Selected Subpopulations

**Figure 2:**
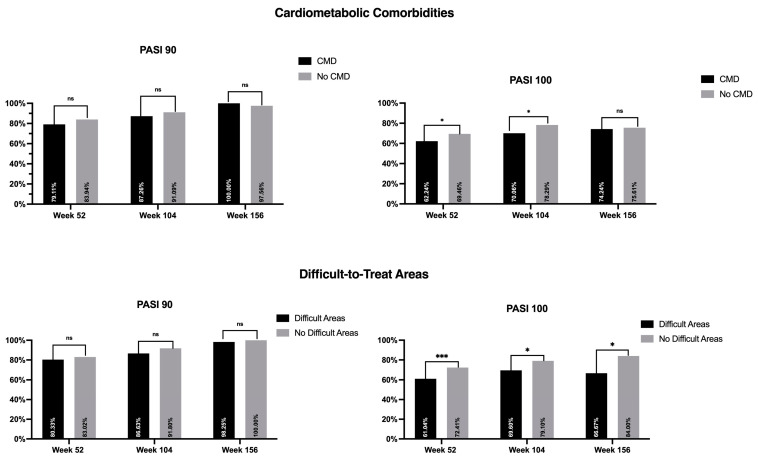
Impact of cardiometabolic comorbidities and involvement of difficult-to-treat areas on PASI 90 and PASI 100. PASI: Psoriasis Area and Severity Index; CMD: cardiometabolic disease; ns: not significant; * *p* < 0.05; *** *p* < 0.001.

The effectiveness of risankizumab was evaluated according to several variables at weeks 52, 104, and 156. All data on the univariate analyses are shown in Table 2 and Table 3. All variables with a *p*-value < 0.2 in the univariate analysis were included in the multivariate analysis. Regarding the involvement of difficult-to-treat areas, from week 52 onwards, patients without difficult-to-treat areas achieved better results in terms of PASI 100, while no differences were seen regarding the other outcomes (Figure 2). In particular, the only negative predictor of PASI 100 at week 156 in our study was the involvement of difficult sites.

In our study, patients with concomitant CMD achieved worse outcomes up to week 104, especially in terms of PASI 100. At week 156, we observed no significant differences between the two categories (Figure 2).

**Figure 3:**
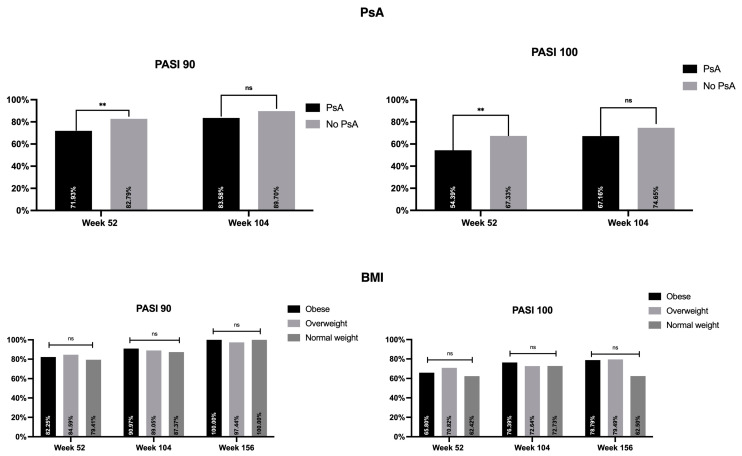
Impact of BMI and PsA on PASI 90 and PASI 100. PASI: Psoriasis Area and Severity Index; BMI: body mass index; PsA: Psoriatic Arthritis; ns: not significant; ** *p* < 0.01.

Interestingly, patients with concomitant diagnosis of PsA tended to respond worse at week 52 compared with those without PsA (Figure 3). No differences were observed at week 104, while the analysis was not performed at week 156 due to the limited sample size of patients with PsA. In terms of BMI, no differences were detected between the three categories starting from week 52 onward.

### 3.4. Multivariate Logistic Regression

We evaluated the predictors of PASI 90 and PASI 100 in the long term by performing a multivariate analysis including all variables with a *p*-value < 0.2 at the univariate analysis. At week 52, we found that patients with PsA had a lower probability of reaching PASI 90 (OR 0.54, C.I. 0.34–0.84, *p* < 0.01). There were multiple variables associated with a lower PASI 100 response, including CMD (OR 0.54, C.I. 0.38–0.77, *p* = 0.001) and involvement of difficult-to-treat areas (OR 0.56, C.I. 0.41–0.76, *p* ≤ 0.001). After two years of treatment, no significant differences were observed in terms of PASI 90, while PASI 100 was negatively associated with CMD (OR 0.61 C.I. 0.42–0.90, *p* < 0.05) and with the involvement of difficult sites (OR 0.59 C.I. 0.40–0.88, *p* < 0.01). At week 156, no variables had a significant impact on PASI 90, and only one was detected for PASI 100. Thus, multivariate logistic regression was not performed at this time point. Complete data regarding the multivariate analysis are shown in Table 2 and Table 3.

### 3.5. Long-Term Effectiveness of Risankizumab on Difficult-to-Treat Areas

**Figure 4:**
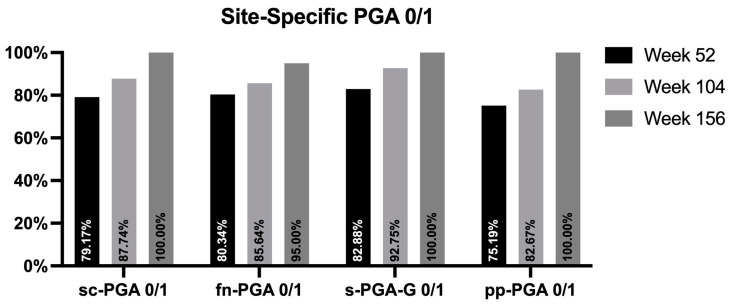
Percentage of patients achieving sc-PGA, pp-PGA, sPGA-G, and f-PGA of 0 or 1 (clear and almost clear) at weeks 52, 104, and 156. sc-PGA: scalp-specific Physician’s Global Assessment; pp-PGA: palmoplantar PGA sPGA-G: static Physician’s Global Assessment of Genitalia; fn-PGA: fingernail PGA.

We conducted a subanalysis on 535 patients who presented with the involvement of at least one difficult-to-treat area and completed at least one year of treatment. Two hundred ninety patients were affected by psoriatic onychopathy, one hundred twenty-nine had palmoplantar psoriasis, two hundred sixteen had scalp involvement, and one hundred eleven suffered from genital psoriasis. After one year of treatment, a site-specific PGA of 0 or 1 was achieved by 79.17%, 75.19%, 82.88%, and 80.34% of patients with the involvement of scalp, palms/soles, genitalia, and fingernails, respectively (Figure 4). Our cohort of patients experienced continuous improvement throughout the study period. After two years of treatment with risankizumab, 87.74%, 82.67%, 92.75%, and 85.64% of patients reached a site-specific PGA of 0 or 1. After three years, complete or almost complete skin clearance was observed in all patients with the involvement of scalp, genitalia, and palms/soles, while 95% of patients with onychopathy achieved the same outcome.

### 3.6. Drug Survival

**Figure 5:**
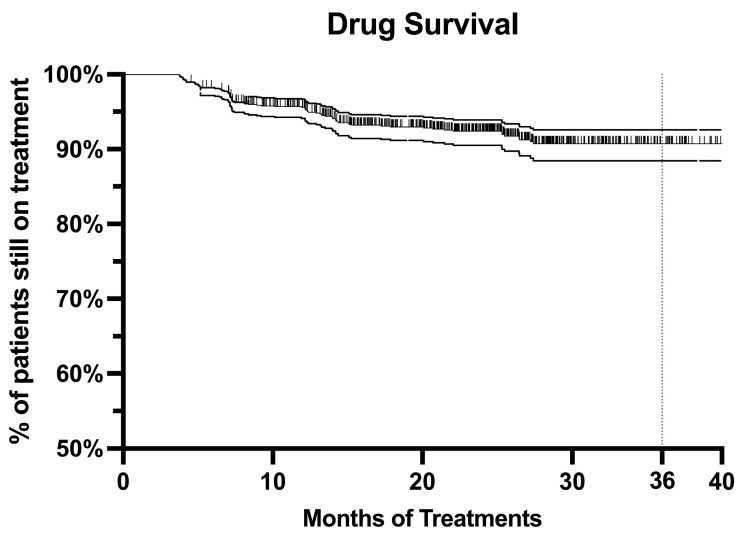
Kaplan–Meier curve of all-cause treatment discontinuation in all patients treated with risankizumab up to 36 months.

The drug survival of risankizumab was evaluated after 36 months using the Kaplan–Meier curve. At three years of therapy, 90.73% (CI 88.44–92.58%) of our patients were still undergoing treatment (Figure 5). The number of patients at risk at months 10, 20, 30, and 36 was 960, 714, 246, and 96, respectively. The log-rank test and Cox regression did not detect any differences in drug survival regarding BMI, gender, age, comorbidities, involvement of difficult-to-treat areas, duration of disease, or previous exposure to biologics. The only predictor was the achievement of PASI 90 at week 16 with a hazard ratio (HR) of 0.26 (CI 0.14–0.47, *p* < 0.001). Eighty-one patients discontinued risankizumab. The main reason for discontinuation was secondary ineffectiveness (48 patients), followed by primary ineffectiveness (18). The mean time to discontinuation due to secondary ineffectiveness was 13.98 months (minimum 5.17 months, maximum 27.4 months). Three patients discontinued risankizumab because of pregnancy. Five patients decided to stop the therapy because of persistent complete skin clearance. The remaining seven patients experienced an AE that led to discontinuation.

### 3.7. Safety

Risankizumab did not show new significant safety findings in our study, as detailed in Table 4. The most frequently reported AEs were nasopharyngitis, upper respiratory tract infections, headache, and diarrhea. Despite fourteen patients with serological evidence of viral hepatitis, one patient with HIV, and ten with positive Quantiferon test results, no reactivation of infections was observed in our experience. Regarding AEs leading to discontinuation, three patients received a cancer diagnosis. One patient developed prostate adenocarcinoma after 16 weeks of treatment. The second case was a fifty-year-old woman who received a diagnosis of ductal breast cancer after 18 weeks of treatment, while the last one was a male heavy smoker who developed lung cancer after 24 weeks of treatment with risankizumab. Three other patients experienced a flare of atopic dermatitis, and one reported an injection-site reaction, leading the clinician to switch the treatment.

## 4. Discussion

Our observational study confirms the effectiveness of risankizumab in a real-life setting, providing additional data on the safety and persistence of the treatment after three years. Our patients had comparable characteristics to those enrolled in clinical trials regarding gender, mean age, and mean BMI. However, in our study, bio-experienced patients were more represented (42.60% versus 34% in the UltIMMa-1 trial), and a significant percentage of them had previous exposure to anti-IL-17, which was under-represented in clinical trials [9]. Mean PASI at baseline was higher in the two clinical trials because of the strict inclusion criteria of the protocols [9]. We observed a continuous improvement throughout the three years of the study regarding all effectiveness endpoints. At week 52, we observed comparable or slightly higher effectiveness with the phase-3 clinical trials UltIMMa-1 and UltIMMa-2 in terms of PASI90 and PASI100 [9].

Higher body weight has been associated with lower clinical response to biological treatments in several studies because of greater drug clearance and volume of distribution in obese patients, resulting in lower systemic exposure [17,18]. In our study, risankizumab showed comparable responses among the three BMI classes, confirming data from real-life studies and subgroup analyses from the IMMerge study [13,19]. Similar effectiveness, regardless of BMI, has also been observed for the other two IL-23 inhibitors [20,21]. However, the presence of at least one CMD in our study was a predictor of lower response to the treatment at weeks 16, 28, 52, and 104, with comparable results to those observed by Adamczyk et al. [22].

In the multivariate analysis, the involvement of difficult-to-treat areas was associated with lower effectiveness in the short term, as expected. However, from week 52 onwards, these differences were no longer recorded, except for PASI100 at week 52, which could be explained by a minimal residual PASI in these areas [13,19,22]. In our study, risankizumab was rapidly effective, particularly in patients with genital psoriasis, which improved patient well-being [23]. Our results support the use of risankizumab in patients involving difficult-to-treat areas due to its high effectiveness in the long term. In our study, risankizumab showed excellent performance in the treatment of difficult-to-treat areas. Our findings are consistent with a recent one-year study by Orsini et al. [24], who observed, after one year of treatment, a site-specific PGA of 0 or 1 in 97.58%, 95.28%, 100%, and 82% of patients involving scalp, palms/soles, genitalia, and nails, respectively.

Risankizumab showed excellent drug survival, as more than 90% of our patients were still on treatment after 36 months. Interestingly, none of the variables previously associated with the higher drug survival of biological treatments were found to be significant in our experience [25,26]. The only exception was represented by patients with an excellent response (meaning those who achieved PASI90 at week 16), who were more likely to still be undergoing treatment after three years.

Risankizumab did not show any new significant safety findings. In patients with previous evidence of exposure to tuberculosis or viral hepatitis, no signs of reactivations were demonstrated at periodical blood examinations or pulmonological or hepatological visits [27,28,29,30]. According to the oncologists, the three patients who received a cancer diagnosis during the treatment discontinued the drug despite no causality nexus being demonstrated. Interestingly, in our study, no cerebrovascular accidents (CVA) were observed after three years of treatment, calling into question two recent reports that detected a disproportionate reporting of CVA with risankizumab [31,32]. According to a recent network meta-analysis by Mattay SS et al. [33], which evaluated 3528 studies on patients with immune-mediated inflammatory disorders treated with biologics or JAK-inhibitors, there was no higher risk of major adverse cardiovascular events (MACE) for those receiving IL-23 inhibitors. Differently, anti-IL-12/23, JAK-inhibitors, and anti-TNF-alfa were associated with a higher risk of MACE compared with placebos, regardless of the underlying inflammatory disease [33].

Our study has a few limitations, represented by its retrospective nature, the absence of a control group, and the heterogeneity of clinical evaluations from different clinicians. In addition, the number of reported AEs was probably underestimated, as in clinical practice it is uncommon for patients to report any mild AEs. However, the main strength of our study is the largest cohort of real-life unselected patients to date treated with an IL-23 inhibitor for plaque psoriasis. Other relevant strengths are the most extended follow-up to date in a real-world setting (156 weeks) and the analysis of several subpopulations of patients.

## 5. Conclusions

This multicenter observational study confirmed the effectiveness and safety of risankizumab in a real-life cohort of patients with plaque psoriasis, with a continuous improvement throughout the 3-year study period regarding all effectiveness endpoints. Risankizumab showed comparable effectiveness across all patient subgroups. The involvement of difficult-to-treat areas, the concomitant diagnosis of PsA, and high BMI did not impact the effectiveness of risankizumab in the long term. In particular, risankizumab showed effectiveness in the subgroup of patients involving difficult-to-treat areas. These initial findings are consistent with post hoc analyses of clinical trials. Regarding safety, no new findings emerged from our long-term observation, supporting the use of risankizumab in a wide cohort of patients.

## Figures and Tables

**Table 1:** Characteristics of the patients at baseline.

Number of Patients	1047
	**N (%)**
Male	705 (67.34%)
PsA	127 (12.13%)
At least one difficult-to-treat site *	623 (59.50%)
At least one cardiometabolic comorbidity	535 (51.10%)
Arterial hypertension	293 (29.98%)
Obesity (BMI ≥ 30)	256 (24.45%)
Type II diabetes mellitus	124 (11.84%)
CVD	56 (5.35%)
Hypercholesterolemia	118 (11.27%)
Bio-naïve	601 (57.40%)
	Mean ± SD
Age, years	51.37 ± 14.95
PASI at baseline	15.73 ± 7.62
BMI	28.05 ± 5.66

SD: standard deviation; BMI: body mass index; PsA: psoriatic arthritis; PASI: Psoriasis Area and Severity Index; mPASI: mean PASI. * Including scalp, palms/soles, genitalia, and nails.

**Table 2:** Univariate and multivariate analyses of predictors (PsA, difficult sites, and CMD) correlated with clinical response to risankizumab after 52 weeks of treatment.

	**PsA**	**No PsA**	**Univariate Analysis**	**Multivariate Analysis**
			***p*-Value**	**OR [CI 95%]; *p*-Value**
PASI 90 w52	82/114 (71.93%)	664/802 (82.79%)	**0.005**	**0.54 [0.34–0.84]; 0.006**
PASI 100 w52	62/114 (54.39%)	540/802 (67.33%)	**0.006**	**0.61 [0.40–0.92]; 0.019**
PASI 90 w104	56/67 (83.58%)	453/505 (89.70%)	0.13	0.61 [0.30-1.25]; 0.18
PASI 100 w104	45/67 (67.16%)	377/505 (74.65%)	0.19	0.73 [0.42-1.27]; 0.27
	**Difficult Areas**	**No Difficult Areas**	**Univariate Analysis**	**Multivariate Analysis**
			***p*-Value**	**OR [CI 95%]; *p*-Value**
PASI 90 w52	433/539 (80.33%)	313/377 (83.02%)	0.30	NA
PASI 100 w52	329/539 (61.04%)	273/377 (72.41%)	**<0.001**	**0.56 [0.41–0.76]; <0.001**
PASI 90 w104	285/328 (86.89%)	224/244 (91.80%)	0.063	0.58 [0.33–1.02]; 0.06
PASI 100 w104	229/328 (69.82%)	193/244 (79.10%)	**0.013**	**0.59 [0.40–0.88]; 0.009**
PASI 90 w156	56/57 (98.25%)	50/50 (100.00%)	0.35	NA
PASI 100 w156	38/57 (66.67%)	42/50 (84.00%)	**0.039**	**0.38 [0.15–0.97]; 0.043**
	**CMD**	**No CMD**	**Univariate Analysis**	**Multivariate Analysis**
			***p*-Value**	**OR [CI 95%]; *p*-Value**
PASI 90 w52	375/474 (79.11%)	371/442 (83.94%)	0.061	0.73 [0.52-1.02]; 0.07
PASI 100 w52	295/474 (62.24%)	307/442 (69.46%)	**0.021**	**0.54 [0.38–0.77]; 0.001**
PASI 90 w104	274/314 (87.26%)	235/258 (91.09%)	0.15	0.63 [0.36-1.09]; 0.1
PASI 100 w104	220/314 (70.06%)	202/258 (78.29%)	**0.026**	**0.61 [0.42–0.90]; 0.013**
PASI 90 w156	66/66 (100.00%)	40/41 (97.56%)	0.20	NA
PASI 100 w156	49/66 (74.24%)	31/41 (75.61%)	0.87	NA

PASI: Psoriasis Area and Severity Index; OR: odds ratio; CI: confidence interval; NA: not applicable (multivariate analysis was not performed for these variables, as the *p*-value at univariate analysis was ≥0.2); CMD: cardiometabolic diseases. Values in bold are statistically significant.

**Table 3:** Univariate and multivariate analyses of obese, overweight, and normal-weight patients after 52 weeks of treatment.

	BMI ≥ 30	25 ≤ BMI < 30	BMI < 25	Univariate Analysis	Multivariate Analysis
				*p*-Value	OR [CI 95%]; *p*-Value
PASI 90 w52	190/231 (82.25%)	258/305 (84.59%)	243/306 (79.41%)	0.25	NA
PASI 100 w52	152/231 (65.80%)	216/305 (70.82%)	191/306 (62.42%)	0.087	1.57 [0.98–2.24]; 0.061
PASI 90 w104	131/144 (90.97%)	179/201 (89.05%)	173/198 (87.37%)	0.58	NA
PASI 100 w104	110/144 (76.39%)	146/201 (72.64%)	144/198 (72.73%)	0.69	NA
PASI 90 w156	33/33 (100.00%)	38/39 (97.44%)	32/32 (100.00%)	0.43	NA
PASI 100 w156	26/33 (78.79%)	31/39 (79.49%)	20/32 (62.50%)	0.20	NA

BMI: body mass index; PASI: Psoriasis Area and Severity Index; OR: odds ratio; CI: confidence interval; NA: not applicable (multivariate analysis was not performed for these variables, as the *p*-value at univariate analysis was ≥0.2).

**Table 4:** Safety profile of risankizumab throughout the study.

Adverse Events	Patients, N (%)
Rhinopharyngitis	21 (2.01)
Headache	15 (1.05)
Upper respiratory tract infections	13 (1.24)
Diarrhea	6 (0.57)
Neoplasm diagnosis	3 (0.29)
Flare of atopic eczema	3 (0.29)
Injection-site reaction	2 (0.19)
AEs leading to discontinuation	7 (0.67)
Total	70 (6.69)

AE: adverse event.

## Data Availability

Additional data supporting the findings of this manuscript are available on reasonable request to the corresponding author.
